# En route to metal-mediated and metal-catalysed reactions in water

**DOI:** 10.1039/c8sc04271c

**Published:** 2018-11-05

**Authors:** Feng Zhou, Chao-Jun Li

**Affiliations:** a Department of Chemistry , FRQNT Center for Green Chemistry and Catalysis , McGill University , Montreal , Quebec H3A 0B8 , Canada . Email: cj.li@mcgill.ca

## Abstract

This perspective report presents the key approaches for the development of various organometallic reactions in aqueous media. In view of future sustainability, the efficient use of natural resources, such as renewable biomass-based feedstocks, constitutes an important aspect for sustainable chemical industry. The exploration and discovery of efficient organometallic reactions or equivalents in water enrich the toolbox of organic chemists for the direct conversion of biomass-derived feedstocks into high-valued chemicals and the direct modification of biomolecules in their native aqueous environment, which contributes to future sustainability.

## Abstract 2

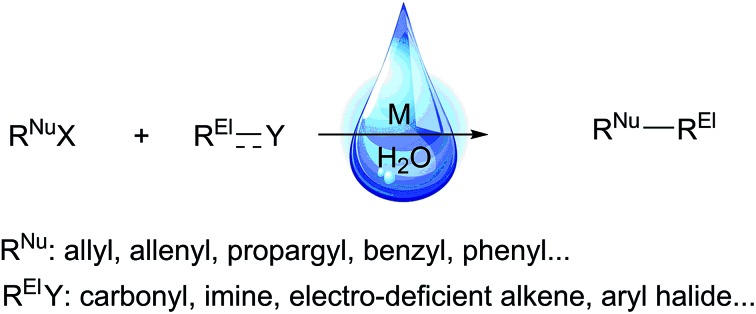
This perspective report presents the key approaches for the development of various organometallic reactions in aqueous media.

## Introduction

Carbon–carbon bond formation plays the central role in synthetic organic chemistry. For polar routes, such transformations necessarily involve carbon nucleophiles and electrophiles.^1^ One class of the most commonly used nucleophiles is organometallic compounds. Since the discovery of alkylzinc compounds by Frankland from the reaction of iodoalkanes with metallic zinc,^2^ organometallic reagents serving as nucleophiles for organic syntheses have been rapidly evolved both in scope and application.^3^–^7^ The early discoveries of Reformatsky,^8^ Barbier,^9^ Grignard,^10^ and Gilman^11^ are among the important milestones in the development of classical organometallic reactions. Subsequently, there have been great progresses in the use of alkali^12^ and other metals since the 1930s. Recently, organometallic reactions catalysed by transition metals have become increasingly important in the synthesis of organic molecules,^13^ underscored by three Nobel Prizes: for palladium-catalysed cross-coupling in 2010,^14^–^16^ olefin metathesis in 2005 17–19 and asymmetric catalysis in 2001 20–22 within the last decade.

In spite of the enormous progress being made, there are obvious shortcomings for classical organometallic reactions in terms of chemical sustainability, such as the extensive use of organic solvents, stoichiometric metals, moisture and functional group intolerances, which are partially ascribed to the limitation of the historical development of classical reactions from fossil based feedstocks^23^ bearing no functional groups and being insoluble in water. Accordingly, classical organic reactions were intuitively developed in fossil originated organic solvents. With the increasing concerns of the depletion of non-renewable fossil resources and the environmental deterioration,^24^ developing products from renewable resources and improving resources/energy utilization efficiency are the key measures towards future chemical sustainability.^25^

Biomass, as a sustainable and renewable feedstock provided by nature could be a practical alternative towards future chemicals.^26^–^28^ In contrast to the fossil-based feedstocks, biomass-based ones are generally over-functionalized and often soluble or soluble after depolymerisation in water. As a result, further processing is required to adapt them to the reaction conditions that were initially developed for fossil-based classical organometallic reactions in organic solvents. One of the most common strategies for such purposes is the exhaustive protecting group manipulations, which in turn limiting the possibility of using water as solvent and leading to extra steps and waste *etc.* Conversely, exploration of organometallic reactions directly in water could be a possible solution in terms of various functional groups tolerance and direct chemical modification of biomolecules. Hence, exploring such fundamentally novel chemistry would potentially simplify chemical synthesis significantly, improve synthetic efficiency, provide valuable tools for chemical biology, and contribute to the future chemical sustainability^29^ with respect to atom-economy,^30^ the E-factor^31^ and step-economy.^32^

## General design concept

Most classical organometallic compounds are highly polarized as carbanions, which are also strong bases. Consequently, they are unstable towards active protons (moisture or functional groups) and must be synthesized/used under strictly anhydrous/aprotic conditions. Conceptually, the key to developing successful organometallic reactions in water (aqueous media) is to attenuate or prevent the protonation of carbon–metal bond once the organometallic species is generated (eqn (1)).
1

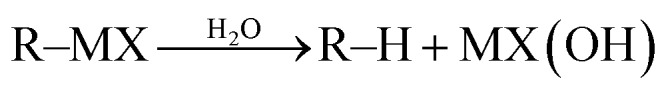




There are several conceivable approaches to achieve this objective. The most straightforward approach is to tune the relative electronegativity^33^ between carbon and metal atoms to form the more covalent C–M bond. A second approach is to design radical pathways, as the strong O–H bond (enthalpy 436 kJ mol^–1^) is very difficult to break homolytically. The third approach is to mimic nature's lipid bilayer membrane^34^ by physically segregating and temporarily stabilizing the organometallic species from water *via* micelle formation using surfactants^35^ or on water strategy.^36^–^38^ The fourth approach is to bypass the stoichiometric organometallic reagents by transition-metal-catalysed organic transformations in water, such as *via* C–H bond activation and hydrazone umpolung.^39^,^40^ This perspective article will illustrate these aspects using the classical nucleophilic additions as examples.

## Various approaches

### Moving towards more covalent C–M bond

**Scheme 1:**
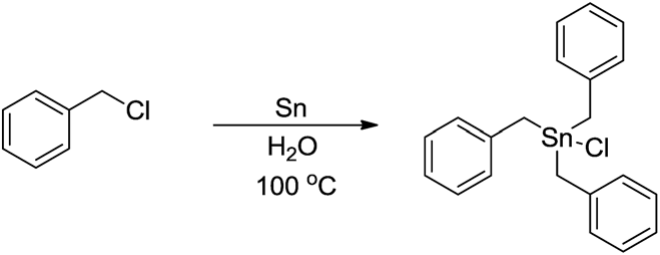
Direct synthesis of tribenzylstannyl halide in water.

The main reason for the necessary anhydrous conditions in classical organometallic reactions was due to the highly polarized and reactive C–M bonds towards water,^3^ especially the organometallic compounds of s-block elements. As C–M bonds become more covalent, such as the organometallic compounds of group 14 and group 15, they have less carbanion character and thus are less prone to acidic proton and relatively more stable in aqueous media. Different from s-block and late p-block organometallic compounds, the ones from group 13 elements take up a special position within the main group due to their moderate reactivity towards water.^41^ Furthermore, certain classes of organometallics remain viable in the presence of water. For example, the preparation of arylmercuric chlorides in aqueous media has been known since 1905.^42^ In the 1960's, tribenzylstannyl halide was prepared in large scale in water (Scheme 1).^43^–^45^ The Wurtz-type reductive coupling of allyl halides proceeded in aqueous alcohol.^46^ These reports opened up new perspectives regarding the metal-mediated organic reactions and it has been increasingly realized that these kinds of reactions can be conducted in water under special circumstances. Indeed, there has been great progress in this research area over the past decades, such as allylation,^47^–^49^ allenylation,^50^,^51^ propargylation,^52^,^53^ benzylation,^54^ phenylation^55^,^56^ *etc.*

**Scheme 2:**
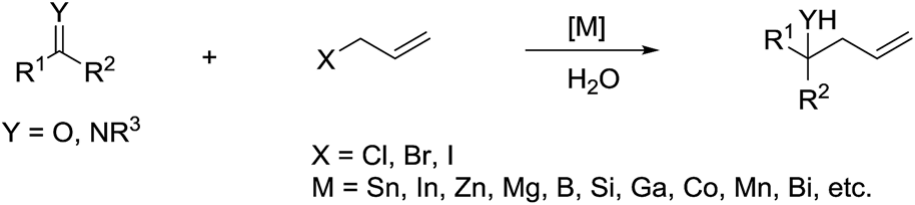
Allylation of carbonyl and imine compounds.

**Scheme 3:**
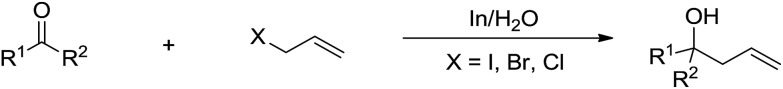
Indium-mediated carbonyl allylation in aqueous media.

The most explored reaction is the allylation of carbonyls and other electrophiles with allyl halides mediated by various metals (Scheme 2),^57^ among which the use of indium to mediate the Barbier–Grignard-type reactions in water reported by Li and Chan received particular attention (Scheme 3).^48^ This was attributed to the fact that on one hand, indium possesses the lowest first ionization potential among the metallic elements near it in the periodic table; and on the other hand, it does not form oxides readily in air and is not sensitive to boiling water or alkali. Consequently, indium was shown to be the most effective for such transformations in water, proceeding smoothly at room temperature without any promoter. This methodology has found wide applications in aqueous synthetic chemistry,^58^–^61^ particularly important in carbohydrate chemistry.

**Scheme 4:**
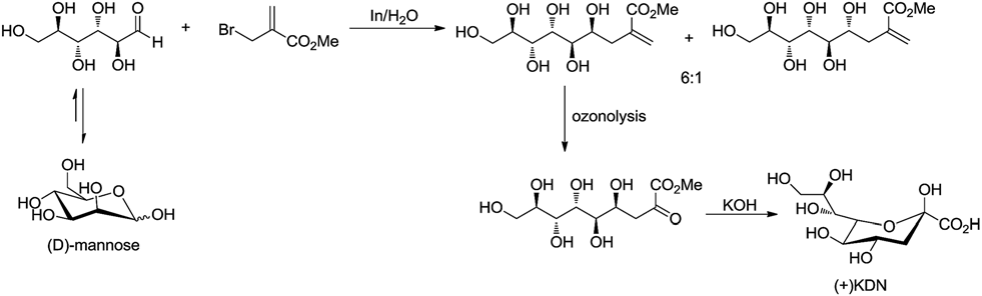
Synthesis of (+)KDN.

**Scheme 5:**
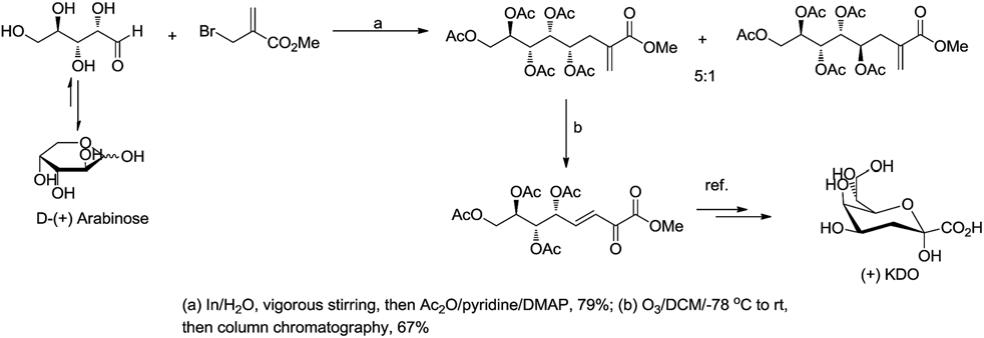
Synthesis of (+)KDO.

**Scheme 6:**
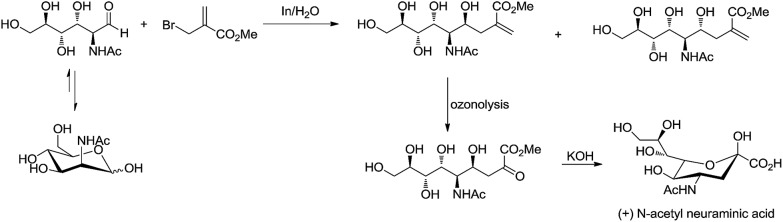
Synthesis of (+)-*N*-acetyl neuraminic acid.

For example, Chan and Li^62^ reported a concise and stereoselective synthesis of (+)-3-deoxy-d-*glycero*-d-*galacto*-nonulosonic acid (KDN) from d-mannose (Scheme 4) and a formal synthesis^63^ of KDO (Scheme 5), whereas Whitesides reported the synthesis of *N*-acetyl-neuraminic acid (Scheme 6)^64^ and other sialic acid derivatives by the method.^65^,^66^ This chemistry has also been extended to six-carbon sialic acid derivatives by Chappell and Halcomb^67^ and the protocol has been further improved by Warwel and Fessner.^68^

**Scheme 7:**
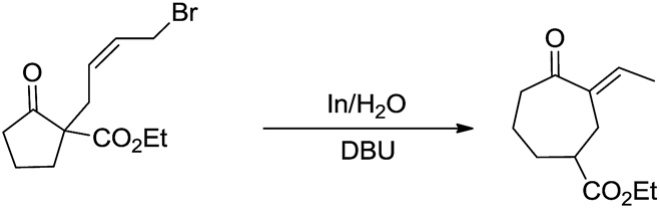
In-mediated carbocyclic ring expansion in water.

Besides being used successfully in carbohydrates chemistry, the metal-mediated allylation has also been applied to the synthesis of other scaffolds such as 1,3-butadienes,^69^ vinyloxiranes, 2-methylenetetrahydrofurans,^70^ trimethylenemethane equivalent,^71^ cyclopentane derivatives.^72^ A carbocyclic ring enlargement methodology was also developed (Scheme 7),^73^,^74^ by using the indium-mediated Barbier-type reaction in water, in which 5-, 6-, 7-, 8-, and 12-membered rings are expanded by two carbon atoms into 7-, 8-, 9-, 10-, and 14-membered ring derivatives respectively. The use of water as a solvent was found to be critical for the success of the reaction and similar ring enlargement in organic solvents was not successful. Such a ring expansion strategy can also be applied to heterocyclic medium ring and^75^ one carbon-ring expansion.^76^

**Scheme 8:**
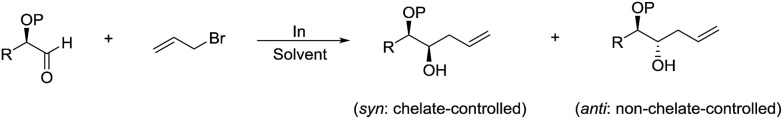
In-mediated diastereoselective allylation of α-oxy aldehydes in water.

The diastereoselectivity of such reaction was studied in detail by Parquette and co-workers, and found that the free hydroxyl derivatives react with excellent diastereofacial control to give significantly heightened levels of *syn*-1,2-diols and *anti*-diols (Scheme 8).^77^ Relative reactivities were determined in the α-series and the hydroxyl aldehyde proved to be the most reactive substrate. This reactivity ordering suggests that the selectivity stems from chelated intermediates. The rate acceleration observed in water can be heightened by initial acidification. Then they reported a variety of diastereoselective allylations in aqueous conditions and their synthetic applications such as a practical alternative to the Knoevenagel reaction of aliphatic aldehydes, the formation of α-methylene-γ-lactones fused to medium and large rings and the intercalation of multiple carbon atoms between the carbonyls of α-diketones.^78^

**Scheme 9:**
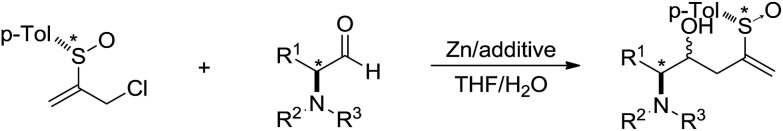
Zinc-mediated Barbier-type diastereoselective allylation of α-amino aldehydes with 2-sulfinylallyl chloride in aqueous media.

In 2002, Delgado and co-workers reported a Barbier-type diastereoselective allylation of α-amino aldehydes with an enantiopure 2-sulfinylallyl building block in aqueous media mediated by zinc (Scheme 9).^79^ High levels of diastereoinduction can be achieved from α-amino aldehydes configurationally related to natural α-amino acids.

**Scheme 10:**
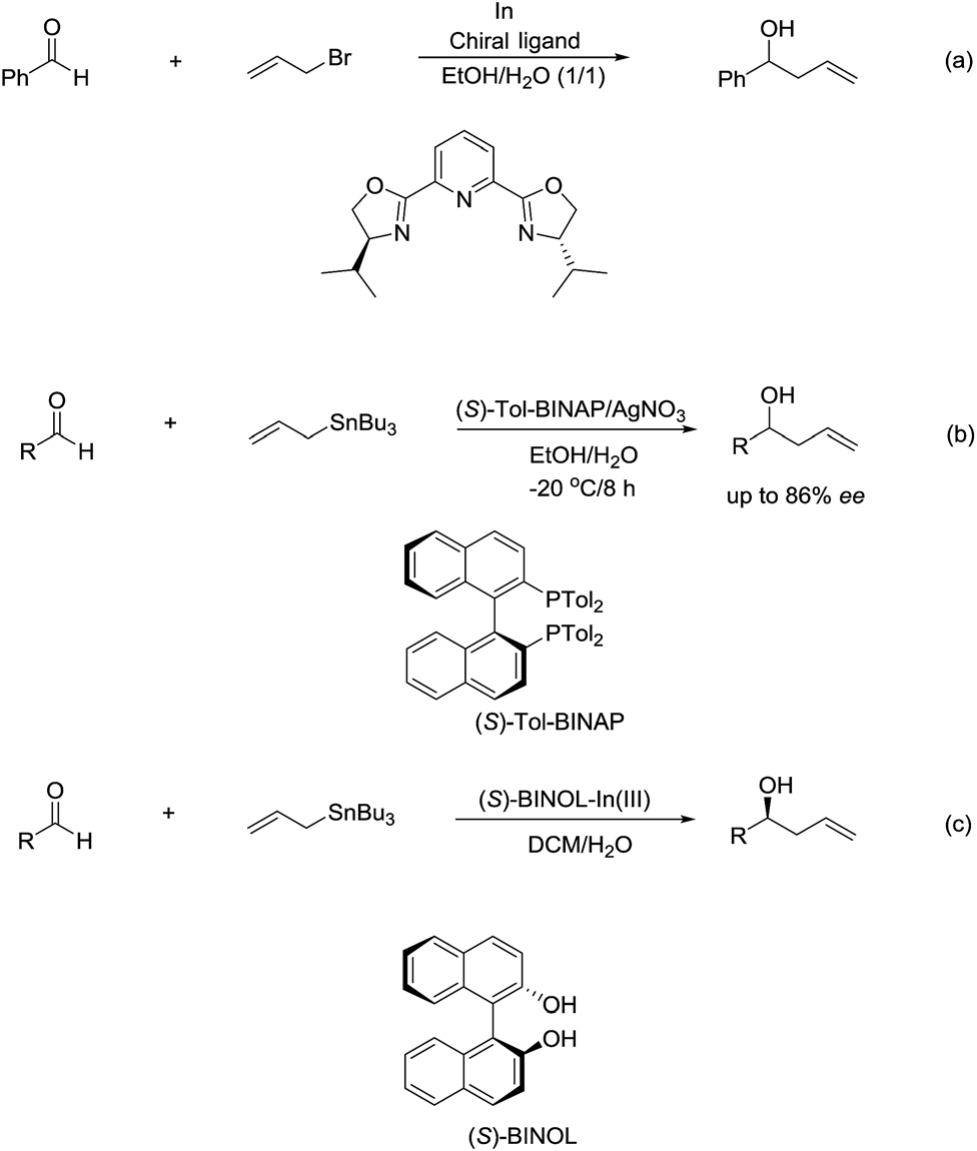
(a) In-mediated enantioselective allylation of aldehydes using chiral pyridine bis(oxazoline) ligand; (b) AgNO_3_/(*S*)-Tol-BINAP-catalysed enantioselective allyation of aldehydes using allyltributylstannane in an aqueous media; (c) In(iii)–(*S*)-BINOL complex catalysed enantioselective allylation of aldehyde using allyltributylstannane in an aqueous media.

The enantioselectivity of such reactions in water was also possible. In 1999, an enantioselective allylation reaction of aldehydes in an aqueous media using chiral pyridine bis(oxazoline) ligand was reported by Loh and co-workers (Scheme 10a).^80^ Subsequently, a AgNO_3_/(*S*)-Tol-BINAP-catalysed enantioselective allylation of aldehydes using allyltributylstannane was achieved by the same group (Scheme 10b).^81^ Later, they reported an enantioselective allylation of aldehyde catalysed by a moisture-tolerant chiral (*S*)-BINOL–In(iii) complex. The allylation of a variety of aromatic, α,β-unsaturated and aliphatic aldehydes resulted in both moderate to good yields and high enantioselectivities (Scheme 10c).^82^

**Scheme 11:**
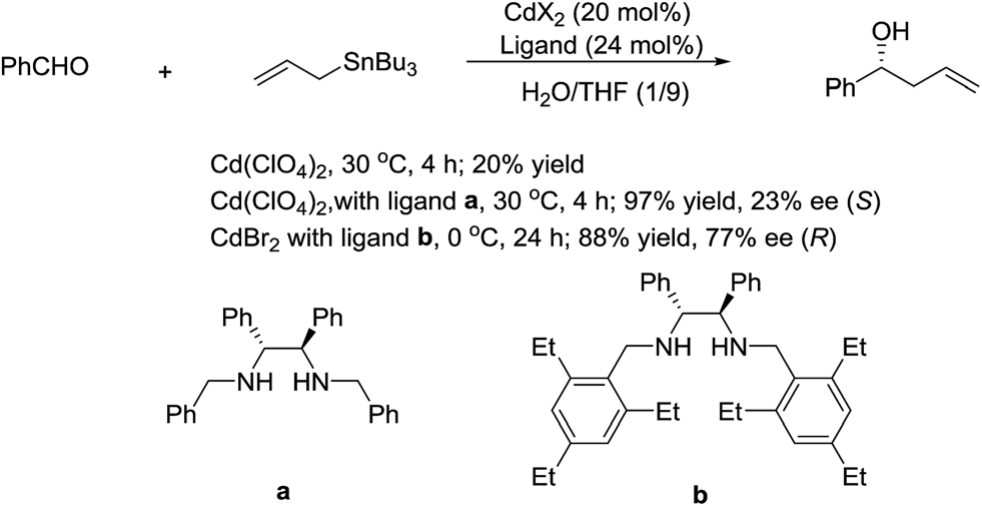
Cd-catalysed asymmetric allylation of aldehyde using allyltributylstannane in an aqueous media.

**Scheme 12:**
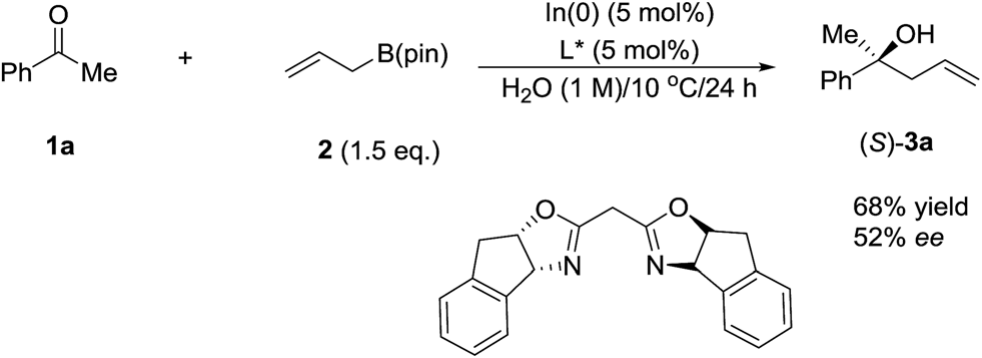
In-catalysed asymmetric allylation of ketone using allyl boronate in water.

In 2003, Kobayashi and co-workers reported a catalytic asymmetric allylation of aldehydes using allyltributyltin in aqueous media *via* the combination of cadmium bromide and chiral diamine ligands. Interestingly, these ligands were found to accelerate the reactions significantly (Scheme 11).^83^ Later, they reported an In-catalysed allylation of ketone with allyl boronates in water (Scheme 12).^84^ Preliminary asymmetric study using chiral bis-oxazoline ligand showed that a moderate enantioselectivity can be obtained.

**Scheme 13:**
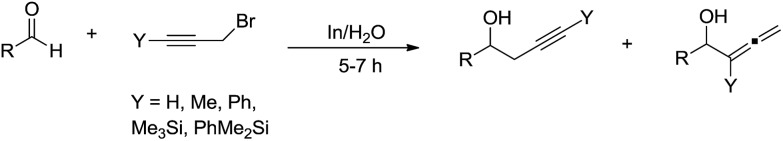
In-mediated propargylation of aldehydes in water.

In addition to allyl halides, structurally related propargyl halides are also effective for such type of reactions. In 1995, Issac and Chan reported their studies of the propargylation of aldehydes in aqueous medium mediated by indium (Scheme 13).^52^ They found that simple prop-2-yn-1-yl bromide reacted with both aliphatic and aromatic aldehydes in water to afford mainly the homopropargyl alcohols. In contrast, when propargyl bromide was γ-substituted, the coupling products were predominantly, or exclusively, the allenylic alcohols. Such couplings also proceed with α-chloropropargyl phenyl sulfide.^85^

**Scheme 14:**
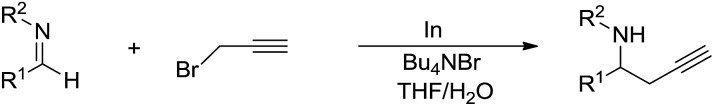
In-mediated propargylation of imines.

The indium-mediated coupling of propargyl bromide with a variety of imines and imine oxides afforded homopropargylamine derivatives in aqueous media under mild conditions (Scheme 14).^86^

Propargylation of glyoxylic oxime ether in the presence of a catalytic amount of palladium(0) complex and indium(i) iodide in aqueous media was also studied.^87^

**Scheme 15:**
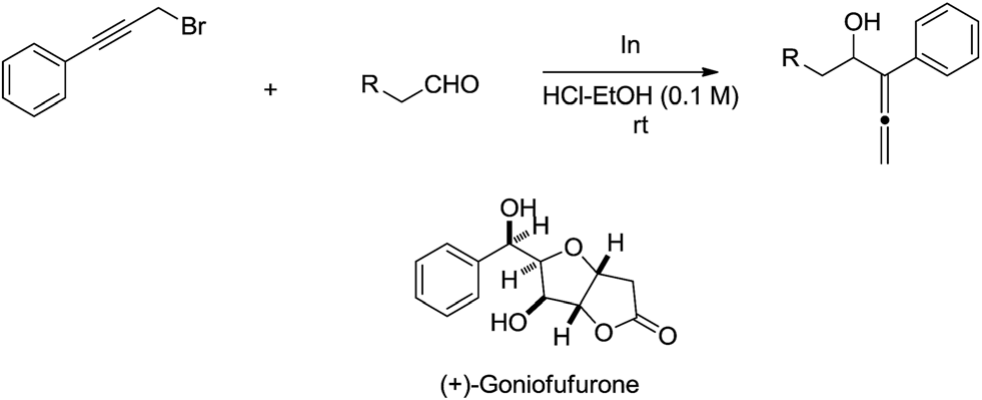
Indium-mediated allenylation in aqueous media and its application to the synthesis of (+)-goniofufurone.

The indium-mediated highly diastereoselective allenylation in aqueous medium was also highly successfully applied to the total synthesis of (+)-goniofufurone (Scheme 15).^50^,^51^

**Scheme 16:**
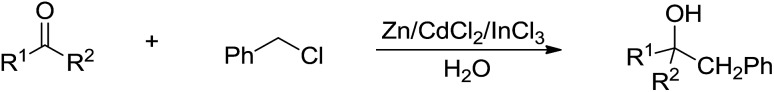
Zn-mediated benzylation of carbonyl compounds in aqueous media in the presence of CdCl_2_ and InCl_3_.

Benzyl halides and allyl(propargyl) halides are structurally similar but have distinctively different chemical reactivities in the aqueous Barbier–Grignard-type reactions. Although tribenzyl and dibenzyltin derivatives have been prepared in aqueous conditions since the 1960s, they do not add onto carbonyls, most likely because it is not possible to form a six-membered cyclic transition state with the carbonyl group in a ‘two-component’ fashion. Still, a zinc-mediated benzylation of carbonyl compounds in aqueous media was reported by Wang and co-workers in 2005 (Scheme 16).^88^

**Scheme 17:**
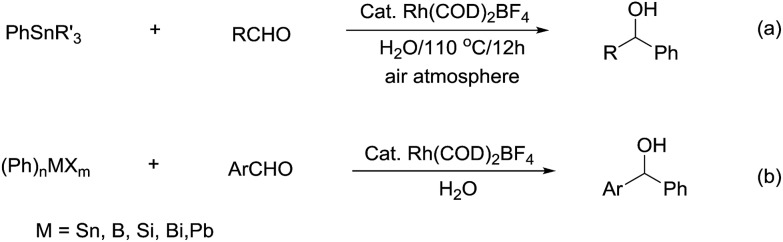
(a) Rh-catalysed carbonyl phenylation in water; (b) the electronic effect on Rh-catalysed carbonyl additions with arylmetallic reagents.

Compared with allyl and propargyl metal species, p-block aryl-metal (and metalloids) (aryl-tin, aryl-boron, aryl-bismuth, aryl-lead, and aryl-silicon) and vinyl-metal species are highly covalent and very unreactive by themselves. However, by utilizing a transition-metal catalyst, such aryl (and vinyl) derivatives become highly reactive, yet stable towards water and protonic functional groups. This strategy has been widely applied for their various cross-coupling reactions with organic halides.^89^ By using a similar strategy, rhodium^90^–^93^ and palladium^94^,^95^ catalysed arylation of carbonyls and (asymmetric) conjugate additions^96^–^101^ with aryl-metal reagents in water and under an air atmosphere are successful (Scheme 17a).^55^ A strong electronic effect on Rh-catalysed carbonyl additions and conjugated additions with various arylmetallic reagents was observed (Scheme 17b).^56^

### Moving towards more radical character

**Scheme 18:**
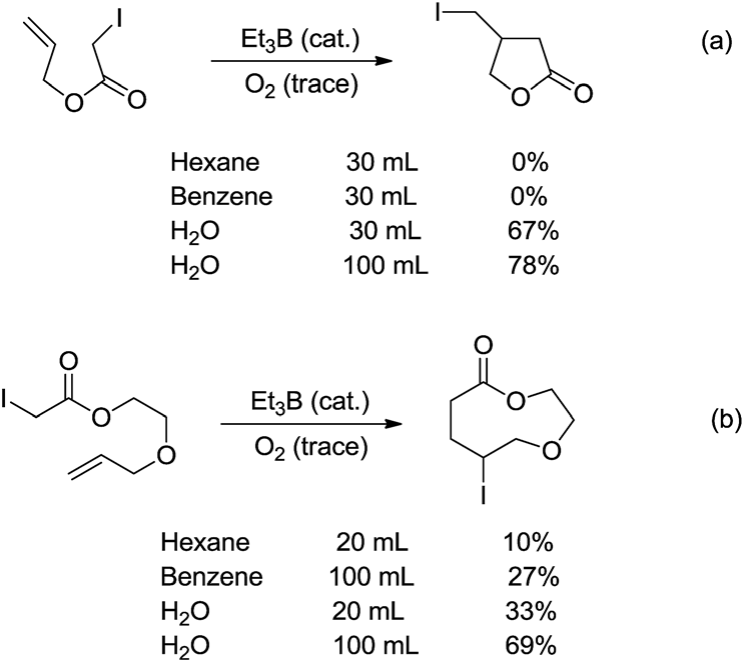
Et_3_B-induced atom transfer radical cyclization in water.

Radicals are neutral species with one unpaired valence electron, which, in contrast to organic anions and cations, make them chemically “stable” towards water. Indeed, the aqueous radical chemistry predominates in biological processes.^102^,^103^ As radicals are nonpolar, their additions to C=C bonds were first realized in water. In 1998, Oshima and co-workers reported that the Et_3_B-induced atom transfer radical cyclization of allyl iodoacetate proceeded much more smoothly in water at ambient temperature than in benzene or hexane (Scheme 18a).^104^ Treatment of the allylic iodoacetate in water with Et_3_B (metalloid) at room temperature for 3 h provided β-iodomethyl-γ-butyrolactone in 67% yield. In contrast, in benzene the desired product was not obtained at all and oligomeric by-products were formed.

The remarkable solvent effect of water was also observed in the case of medium and large ring construction. For example, Oshima and co-workers reported an intramolecular cyclization mediated by Et_3_B in water provided the 9-membered lactone in 69% yield (Scheme 18b). The same reaction carried out in benzene afforded much inferior yield.^105^ Although the exact role of water was not clear at that stage, a hydrogen bonding between water and the carbonyl oxygen could be formed to facilitate the abstraction of iodine, generating the (alkoxycarbonyl)methyl radical. Hydrophobic interaction may also accelerate the cyclization.

**Scheme 19:**
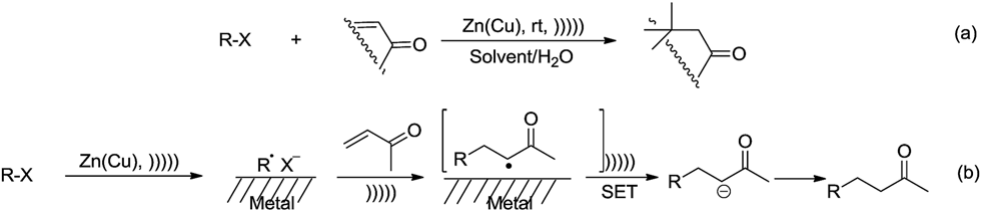
Zinc–copper couple-mediated conjugate additions of alkyl halides to carbonyl compounds in aqueous media and the proposed radical mechanism.

**Scheme 20:**
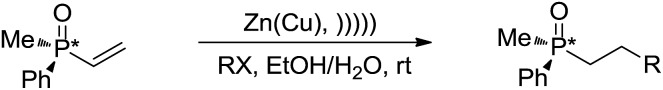
Zinc–copper couple-mediated conjugate addition of alkyl halides to vinylphosphine oxides in aqueous media.

With regard to metal-mediated conjugate additions involving alkyl groups, Luche and coworkers reported that alkyl halides in the presence of zinc-copper couple reacted smoothly with conjugated carbonyl compounds and nitriles to give 1,4-addition products in good yields under sonication conditions in aqueous conditions (Scheme 19a).^106^–^108^ A moderate diastereoselectivity was obtained in those reactions where a mixture of diastereomers could be generated.^109^,^110^ The reactivity of the halides followed the order of tertiary > secondary [right double angle bracket] primary, and iodide > bromide (chlorides did not react). The preferred solvent system was aqueous ethanol. The reaction was proposed to undergo a free radical mechanism occurring on the metal surface under sonochemical conditions (Scheme 19b). Efforts to trap the intermediate intramolecularly only gave a very low yield of the cyclization product.^111^ Similar additions also occurred on vinylphosphine oxides. When optically active vinylphosphine oxide was used, P-chiral alkylphosphine oxide was obtained with retention of configuration (Scheme 20).^112^

**Scheme 21:**
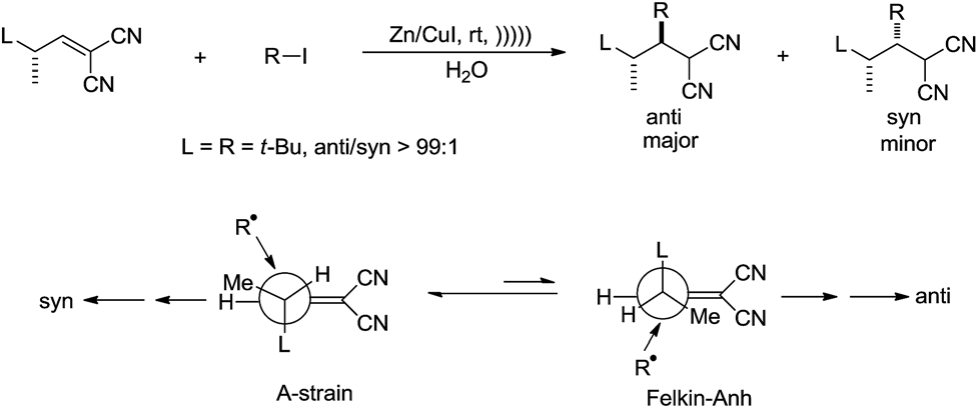
Zinc–copper couple-mediated conjugate addition of alkyl halides and electron-deficient alkenes in water.

Giese and co-workers studied the diastereoselectivity associated with a related addition in water^113^ and found that the anti-isomer was the main product if the attacking radical is bulky (Scheme 21).

The authors rationalized the high diastereoselectivity by proposing that the more stable ‘A-strain’ conformer of the alkene reacts much slower with bulky alkyl radical than the less stable ‘Felkin–Anh’ conformer.

**Scheme 22:**
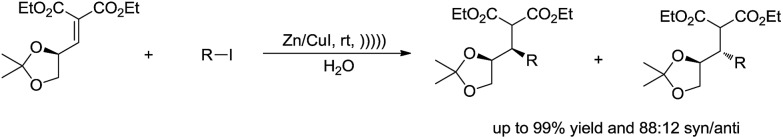
Zinc–copper couple-mediated diastereoselective addition of alkyl iodides and α,β-unsaturated compounds in water.

The diastereoselective ultrasonically induced 1,4-addition of alkyl iodides in the presence of zinc–copper couple to chiral α,β-unsaturated systems in aqueous media was studied by Suarez and co-workers (Scheme 22). They observed that good diastereoselectivities were obtained with the *Z*-isomer while the reactions with the *E*-isomer were non-stereoselective.^114^,^115^

**Scheme 23:**
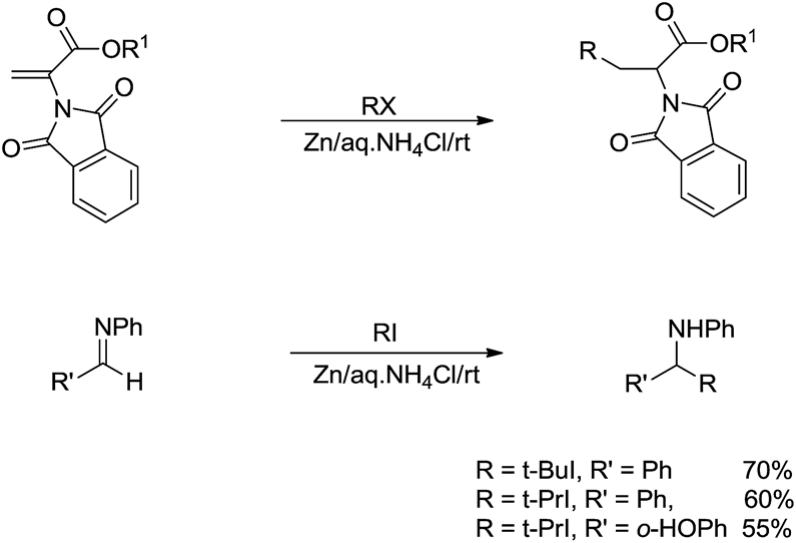
Zinc-mediated conjugate addition of alkyl halides to α-phthalimidoacylate and imine derivatives in aqueous media.

For the more challenging nucleophilic addition of radical-based alkyl-metal species to polar C=X bonds, the key is to prevent the more facile reversed reaction to break the C–C bonds of the radical addition intermediate. One approach is *via* the stabilization of the radical. Thus, in 2002, Li and co-workers reported a zinc-mediated conjugate addition reaction of alkyl halides to α-phthalimidoacylate derivatives and nucleophilic addition to imines for the efficient synthesis of α-amino acid derivatives and amines in the presence of NH_4_Cl in water (Scheme 23). Notably, no reaction was observed in absence of water.^116^

**Scheme 24:**
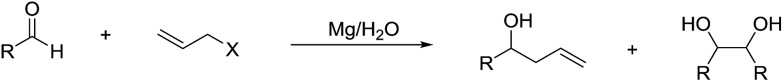
Barbier–Grignard allylation of aldehydes with magnesium in water.

It is noteworthy that Li and co-workers also reported a magnesium-mediated Barbier-type allylation of aldehyde using water as a sole solvent, which proceeds most likely *via* a radical process on the metal surface (Scheme 24).^117^

**Scheme 25:**
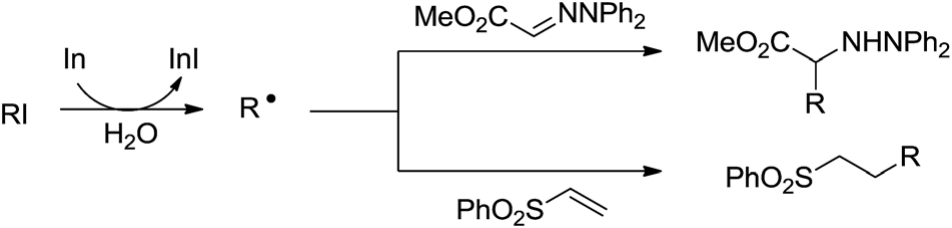
In-mediated alkyl radical addition to imine and phenyl vinyl sulfone derivatives in aqueous media.

In 2002, Naito and co-workers reported an intermolecular alkyl radical addition to imine derivatives and electron-deficient C–C double bond in aqueous media by using indium as a single-electron-transfer radical initiator (Scheme 25).^118^ The one-pot reaction provided a convenient method for preparing α-amino acids.

**Scheme 26:**
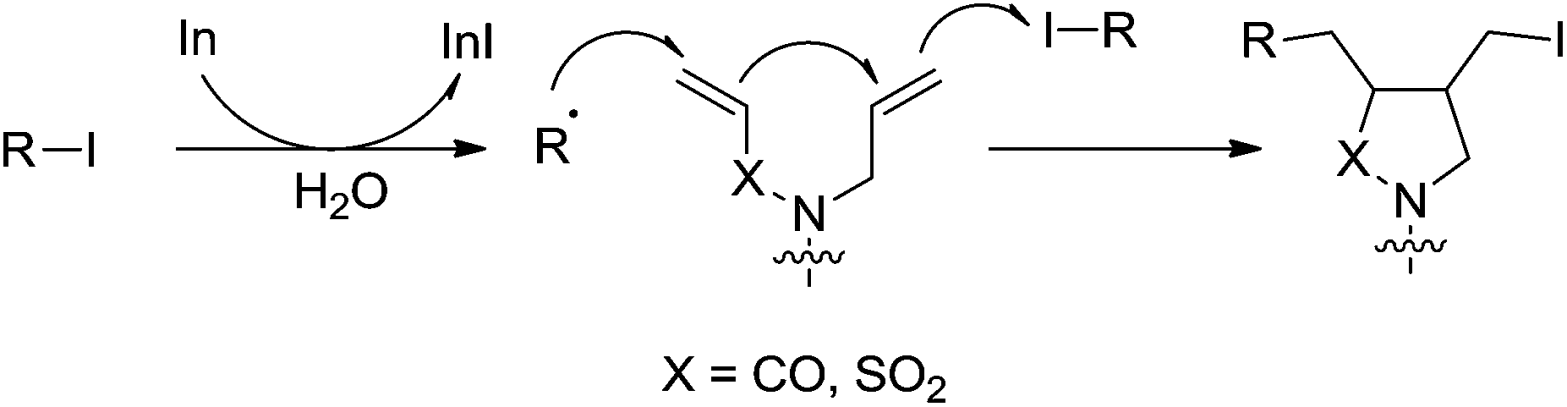
Indium-mediated radical addition–cyclization–trapping cascade reaction in aqueous media.

In 2003, they reported an indium-mediated cascade reaction, in which the addition–cyclization–trapping sequences efficiently generated the cyclized products in aqueous media (Scheme 26).^119^ The substrates bearing vinylsulfonamide and hydrazone proceeded smoothly in aqueous media to provide the functionalized cyclic products.

**Scheme 27:**
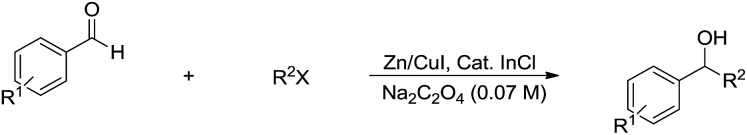
Zinc-mediated Barbier–Grignard-type carbonyl alkylation in water.

For the even more challenging Barbier–Grignard-type carbonyl alkylation using unactivated alkyl halides in water, in absence of radical stabilization, the “reductive Lewis acid” concept was used by Li and co-workers successfully, in which conceptually the addition is accompanied by reduction and “free radical intermediate” is not “free” during the reaction progress (Scheme 27).^120^

**Scheme 28:**
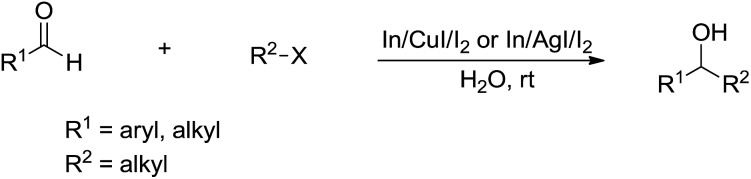
In/CuI or In/AgI-mediated alkylation of carbonyls in water.

In 2008, Loh and co-workers further developed this Barbier–Grignard-type alkylation reaction of aldehydes including aliphatic version using unactivated alkyl halides in water catalysed by In/CuI or In/AgI catalysis (Scheme 28).^121^ The reactions proceeded more efficiently in water than in organic solvent.

**Scheme 29:**
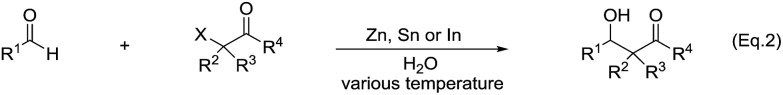
Zn, Sn and In-mediated direct cross-aldol reactions of aldehydes and α-halogen carbonyls.

**Scheme 30:**
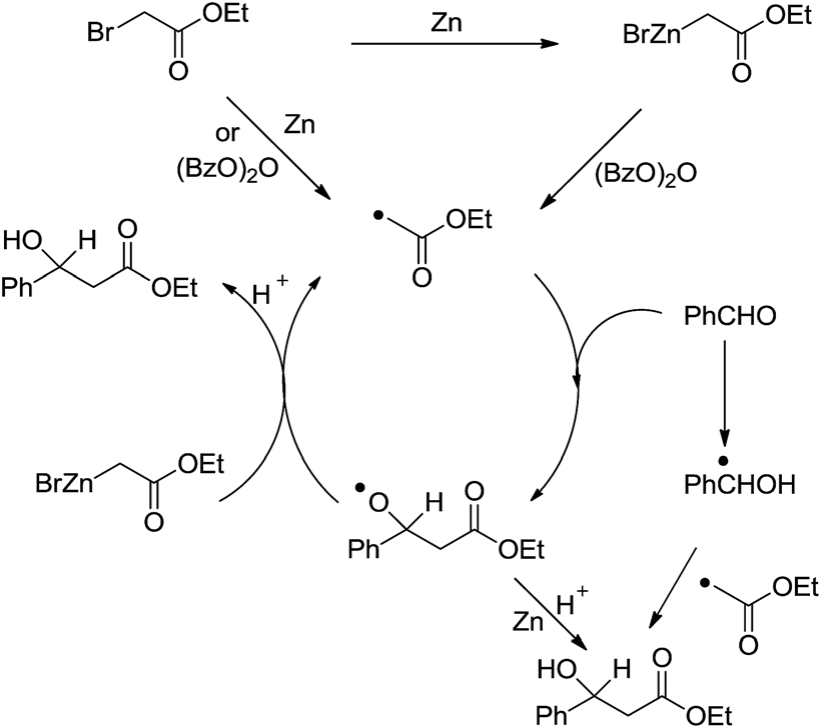
Zinc-mediated Reformatsky-type reaction of bromoacetates and aromatic aldehydes and the proposed radical chain mechanism.

Another type of reactions is the metal-mediated Reformatsky-type reaction. The reaction of a metal with an α-halogen carbonyl compound would generate an organometallic intermediate that can equilibrate between the carbanion form and the enolate form. Like the case of allyl and propargyl, the enolate form would allow the reaction to proceed through a six-membered cyclic transition state and thus could be energetically favorable. Indeed, the reaction of an α-halogen carbonyl compound with an aldehyde in the presence of zinc, tin, or indium in water provided a direct cross-aldol reaction product (Scheme 29).^122^,^123^ While a direct Reformatsky-type reaction occurred in low yields in the case of aromatic aldehydes were used in water mediated by zinc.^124^ Later Lee and co-workers reported that the reactions of aldehydes or ketones with ethyl bromoacetate in the presence of indium promoted by ultrasound afforded β-hydroxyesters in good to excellent yields under mild conditions.^125^ Bieber and co-workers found that even catalytic amounts of benzoyl peroxide or peracids can greatly enhance the reactions of bromoacetates and aromatic aldehydes, giving satisfactory yields. A radical chain mechanism, initiated by electron abstraction from the organometallic Reformatsky reagent, was proposed (Scheme 30).^126^

**Scheme 31:**
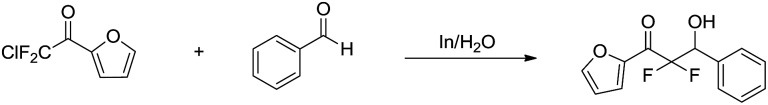
In-mediated synthesis of α,α-difluoro-β-hydroxy ketones in water.

The indium-mediated aqueous Reformatsky reaction was successfully applied to the synthesis of α,α-difluoro-β-hydroxy ketones by Welch and co-workers in 2001 (Scheme 31).^127^

### *Via* temporarily stabilization (surfactant) or on water strategy

One strategy that nature frequently adopts to perform reactions in aqueous media is encapsulation *via* lipid bilayer membranes.^34^ Chemists, in turn, have developed surfactants that can be self-assembled in water to form micelles,^128^ which provide the lipophilic interior to serve, in essence, as the solvent for catalysis. Organic solutes interact with micelles according to their polarity: non-polar solutes are encapsulated in the interior of the micelle, while polar solutes locate themselves at the surface of the micelle and moderately polar molecules would be positioned closer to the polar surface. This compartmentalization of solutes lays the foundation and is responsible for many organic reactions in aqueous media, particularly assisting with organometallic processes, as the lipophilic interior of the surfactants provides the hydrophobic area to temporarily stabilize polar carbon–metal bond and facilitate the chemical transformation, such as in oxidation, reduction and C–C coupling reactions.^129^

**Scheme 32:**
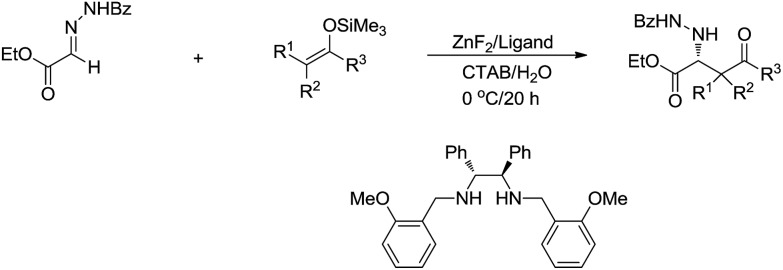
Zinc-mediated asymmetric Mannich-type reactions in water using CTAB as surfactant.

Recently, the application of surfactants for aqueous catalysis has been demonstrated in the development of catalytic organometallic reactions in water. For example, in 2004, Kobayashi and co-workers reported a Zn-mediated enantio and diastereoselective, stereospecific Mannich-type reaction in water, in which the surfactant CTAB (cetyltrimethylammonium bromide) significantly improved the yield (Scheme 32).^130^

**Scheme 33:**
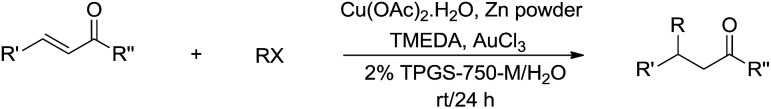
Cu-catalysed and zinc-mediated conjugate additions of alkyl halides to enones in aqueous media using TPGS-750-M as surfactant.

In 2012, Lipshutz and co-workers reported a copper-catalysed conjugate addition of alkyl halides to enones mediated by zinc in aqueous media using TPGS-750-M as surfactant (Scheme 33).^131^ Various cross-coupling reactions catalysed by transition metals in aqueous media using surfactants were also reported by his group.^132^,^133^

**Scheme 34:**
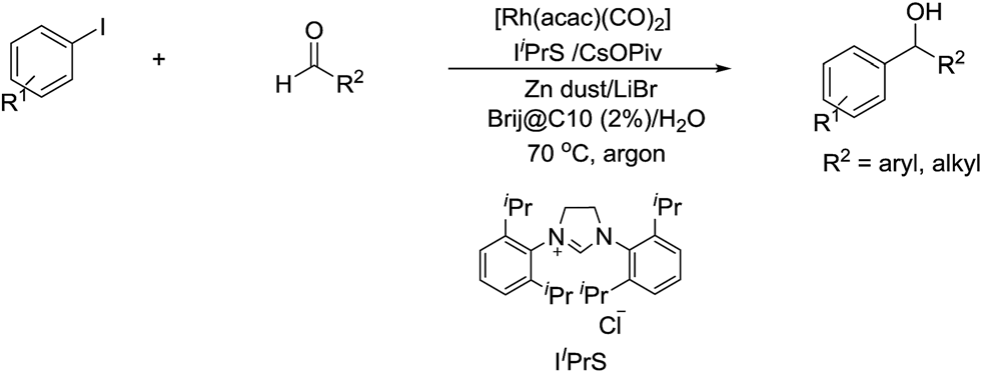
Rh-catalysed and Zn-mediated Barbier–Grignard-type arylation of aldehydes in water using BrijC10 as surfactant.

In 2014, Li and co-workers reported the Rh-catalysed and Zn-mediated Barbier–Grignard-type arylation of aldehydes using unactivated aryl iodides in water, in which the organometallic species could be temporarily stabilized by encapsulation into the lipophilic interior of the surfactant BrijC10 (Scheme 34).^134^

**Scheme 35:**
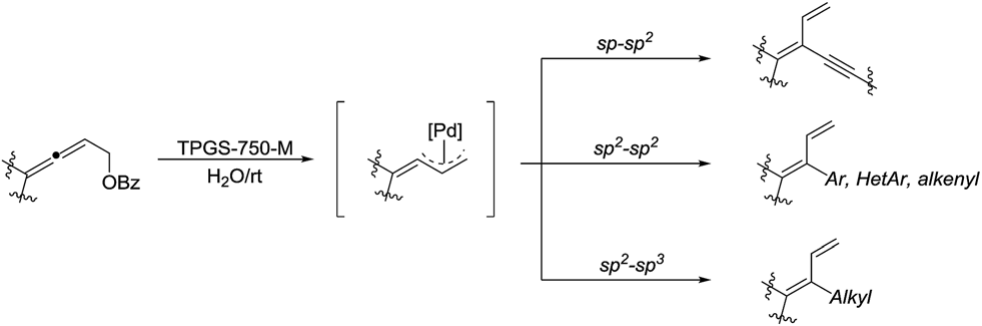
Pd-catalysed cross-coupling of substituted allenic esters for the synthesis of functionalized 1,3-butadienes in water in the presence of the surfactant TPGS-750-M.

In 2018, Lipshutz and co-workers developed an environmentally responsible, mild method for the synthesis of functionalized 1,3-butadienes *via* Pd-catalyzed cross-coupling of substituted allenic esters in water in the presence of the surfactant TPGS-750-M (Scheme 35).^135^ Various sp–sp^2^, sp^2^–sp^2^, and sp^2^–sp^3^ coupling reactions were realized and these transformations tolerated broad functional groups.

**Scheme 36:**
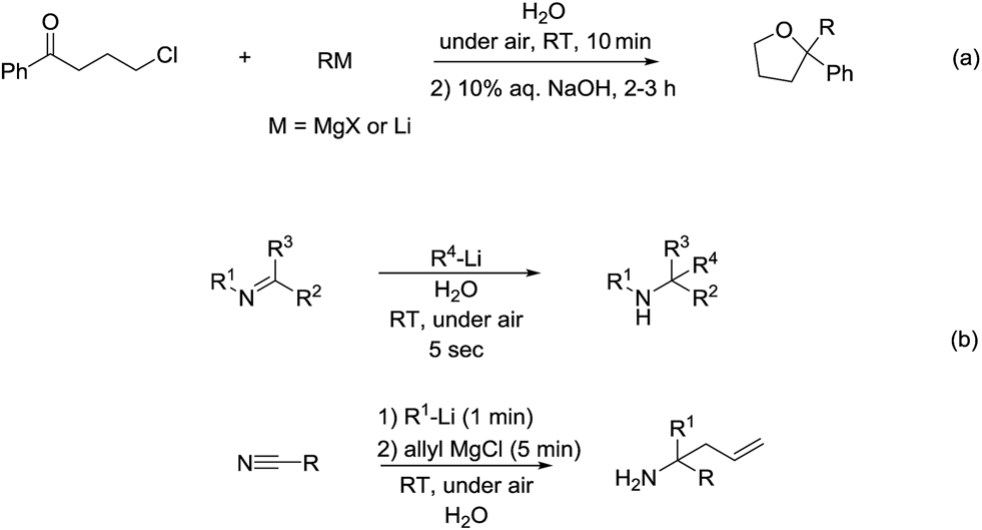
(a) Formation of THF derivatives *via* nucleophilic additions of Grignard reagents to γ-chloroketone using on water strategy; (b) on-water addition of organolithium and organomagnesium reagents to imines and nitriles.

As for the reactivity of polar organometallic compounds on water, recently Capriati and co-workers reported the nucleophilic addition of Grignard reagents and lithium reagents to γ-chloroketone on water at room temperature and under air for efficient formation of THF derivatives, in which water may play an important role as demonstrated by the solvent isotope effect and the control experiments that indicate the strong intermolecular hydrogen bonds jointly with *trans*-phase H-bonding with the substrate, thus (a) shielding the organometallic reagent from competitive protonolysis processes and (b) activating the carbonyl derivative towards nucleophilic addition (Scheme 36a).^136^ Later, they further developed the nucleophilic additions of organolithium and organomagnesium reagents to imines and nitriles using bulk water as a privileged reaction medium, working under air, at room temperature, with vigorous stirring (Scheme 36b).^137^ The significant solvent D/H isotope effect observed for the on-water nucleophilic additions of organolithium compounds to imines suggests the on-water catalysis arises from proton transfer across the organic–water interface. The strong intermolecular hydrogen bonds between water molecules may play a key role in disfavouring protonolysis, which occurs extensively in other protic media such as methanol.^138^,^139^

### Bypass stoichiometric reagents

#### *Via* C–H bond activation

The rising of green chemistry raises the awareness of atom economic and environmentally friendly method development.^140^ In this context, a large number of highly efficient and selective reaction systems have been developed, in which C–H activation catalysed by transition metals is undoubtedly one of the fastest growing areas.^141^–^143^ In view of both synthetic efficiency and future chemical sustainability, the development of the organometallic reactions in water *via* C–H bond activation, generating a transient C–M based intermediate that quickly reacts with electrophiles rather than water in absence of stoichiometric organometallic reagents, becomes attractive.^144^

**Scheme 37:**
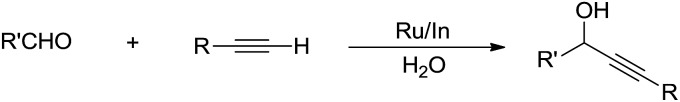
Ru/In co-catalysed alkynylation of aldehydes in water.

In the past two decades, there have been great advances in aqueous C–H bond activation catalysed by transition metals. Since C(sp)–H bonds are the most acidic, hence the easiest to activate in water. In 2002, Li and co-workers reported that a bimetallic Ru–In catalytic system could catalyse alkynylation of aldehydes in water (Scheme 37).^145^

**Scheme 38:**
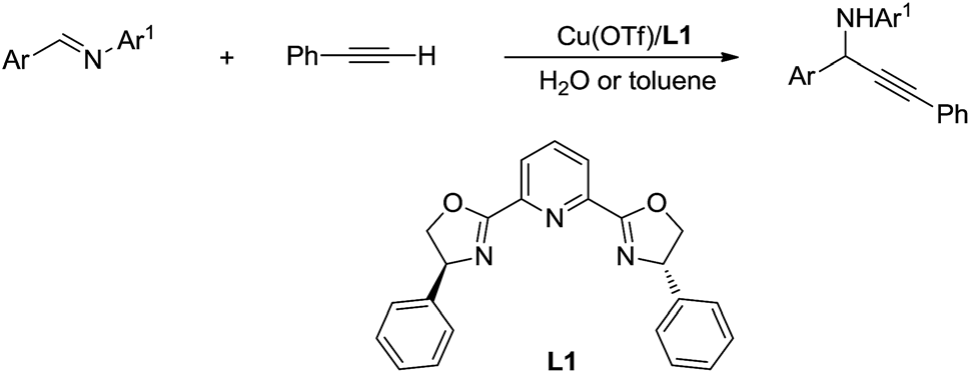
Cu/pyridine-oxazoline catalysed asymmetric addition of phenylacetylene to imine in water *via* C(sp)–H activation.

Then, by using Cu/Ru or Cu/pyridine-oxazoline catalyst, they succeeded the addition and asymmetric addition of arylacetylenes to imines to form propargylamines in excellent yields and enantioselectivity in water (Scheme 38).^146^,^147^ Furthermore a series of transition-metal-catalysed C(sp)–H bond activation reactions in water were reported by his group.^148^,^149^

**Scheme 39:**
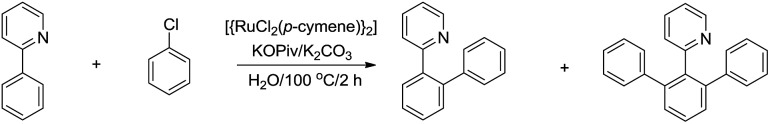
Ru-catalysed *ortho*-phenylation in water *via* C(sp^2^)–H bond activation.

For the less acidic C(sp^2^)–H bonds, one strategy to facilitate their reaction in water is *via* chelation. In 2010, Dixneuf and co-workers reported a Ru-catalysed and pyridine-directed C(sp^2^)–H bond activation in water for efficient *ortho*-phenylation (Scheme 39).^150^ The selectivity of mono-phenylation and bis-phenylations was found to be better in water than in organic solvent.

**Scheme 40:**
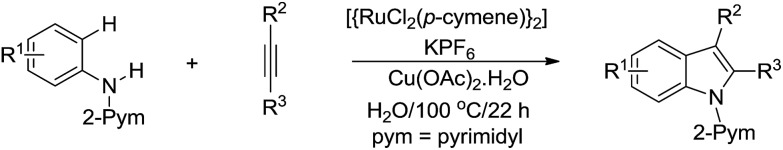
Ru-catalysed tandem cyclization of aniline derivative and alkyne for indole synthesis in water *via* C(sp^2^)–H bond activation.

In 2012, Ackermann and co-workers developed the Ru-catalysed tandem cyclization of aniline derivative and alkyne for efficient indole synthesis *via* C(sp^2^)–H bond activation in water (Scheme 40).^151^

**Scheme 41:**
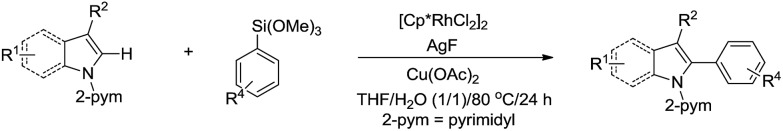
Rh-catalysed 2-phenylation of indole derivatives in water *via* C(sp^2^)–H bond activation.

**Scheme 42:**
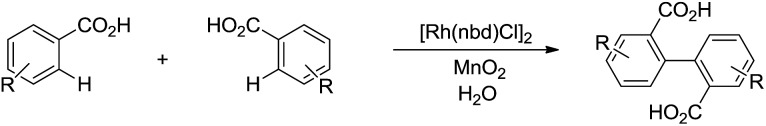
Rh-catalysed homo-coupling of aryl carboxylic acid in water *via* two-fold C(sp^2^)–H bond activation.

In 2014, Loh and co-workers presented the Rh-catalysed and pyrimidine-directed 2-phenylation of indole derivatives *via* C(sp^2^)–H bond activation in water using trimethoxyphenylsilane as phenylation reagent (Scheme 41).^152^ In 2015, Li and co-workers reported the Rh-catalysed homo-coupling of aryl carboxylic acid in water *via* two-fold C(sp^2^)–H bond activation using MnO_2_ as terminal oxidant (Scheme 42).^153^

**Scheme 43:**
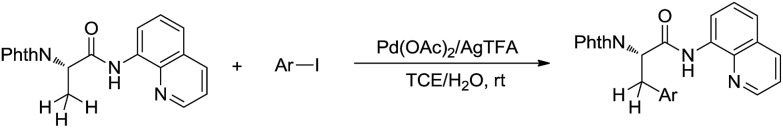
Pd-catalysed *N*-quinolylcarboxamide directed β-arylation of alanine at room temperature *via* C(sp^3^)–H bond activation using water as a co-solvent.

Such a chelation strategy can also be applied towards the least acidic C(sp^3^)–H bonds. For example, in 2014, Chen and co-workers reported a Pd-catalysed *N*-quinolylcarboxamide directed β-arylation of alanine at room temperature *via* C(sp^3^)–H bond activation, in which water is used as a co-solvent (Scheme 43).^154^ This method provided a convenient approach for the synthesis of both natural and unnatural aromatic α-amino acids.

**Scheme 44:**
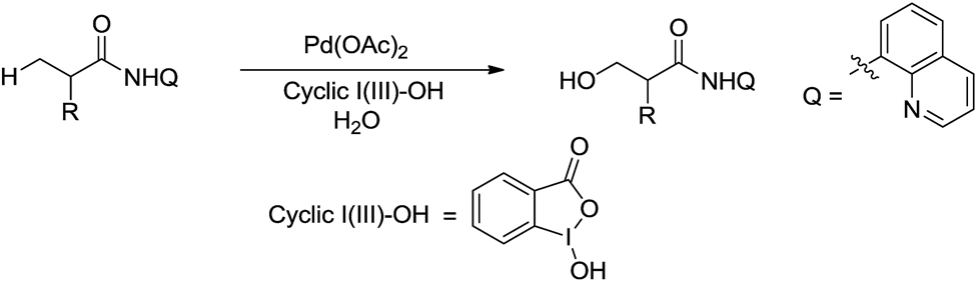
Pd-catalysed β-C(sp^3^)–H bond oxidation of amides using 8-aminoquinoline as directing group in water.

In 2015, Rao and coworkers reported the Pd-catalysed β-C(sp^3^)–H bond oxidation of amides using 8-aminoquinoline as directing group in water (Scheme 44).^155^ Interestingly, the isotope labelling experiment indicates that the oxygen originates from water.

**Scheme 45:**
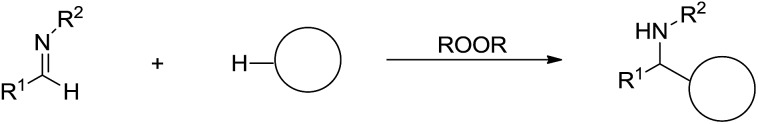
The peroxide-mediated direct addition of cycloalkanes to imines.

An alternative strategy for the C(sp^3^)–H bonds activation is *via* radical process. In 2008, Li and co-workers reported a direct addition of cycloalkanes to imines mediated by peroxide and the reaction tolerates a wide range of functionalities as well as aqueous conditions (Scheme 45).^156^

#### *Via* hydrazone umpolung

**Scheme 46:**
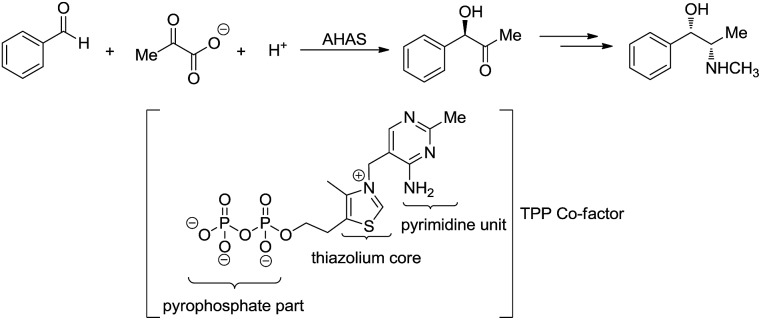
AHAS catalysed acyloin condensation and the synthesis of ephedrine.

**Scheme 47:**
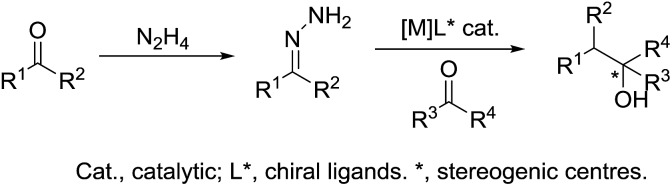
Metal-catalysed umpolung chemistry *via* hydrazone intermediate.

Umpolung is a phenomenon in which the polarity of a functional group is reversed.^157^,^158^ This opens up reactions on a functional group which is otherwise not possible. In nature, numerous enzymes such as acetohydroxy acid pseudoephedrine synthase (AHAS) catalyse both nucleophilic acylation and benzoin condensation reactions in aqueous media *via* umpolung strategy, in which a cofactor thiamine pyrophosphate (TPP) facilitates the catalytic function of these enzymes (Scheme 46).^159^–^161^ Inspired by the biocatalytic methods that use enzymes as catalysts for various C–C bond forming reactions, chemists have successfully developed numerous C–C forming reactions based on umpolung with carbonyls as acyl anion equivalents. Most of those reactions were catalysed by either *N*-heterocyclic carbene (NHC) or cyanide ion.^162^,^163^ Related to organometallic reactions, one attractive approach is the metal-catalysed umpolung chemistry *via* hydrazone intermediate that originates from naturally occurring carbonyls (Scheme 47). This strategy not only improves compatibility towards benign protic solvents and accommodates various functional groups, but also provides an opportunity for enantioselective catalysis when involving chiral ligands.

**Scheme 48:**
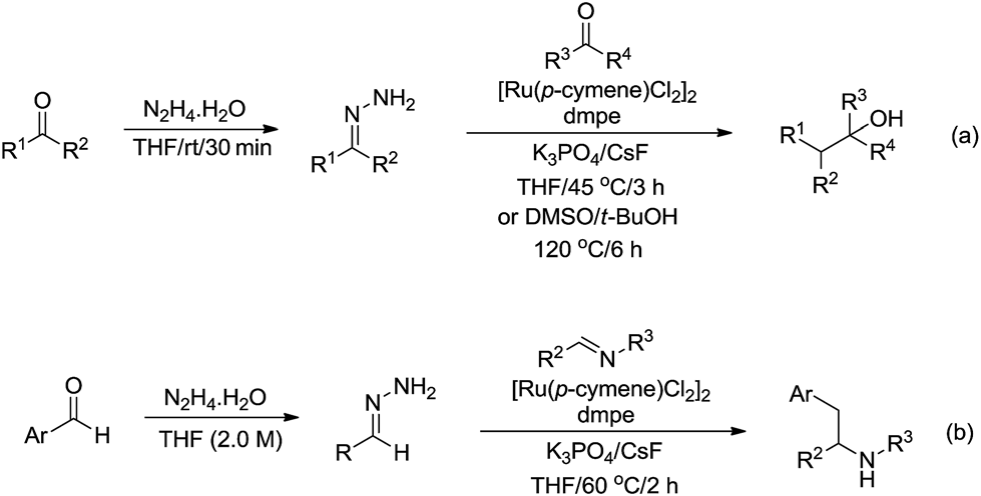
Ru-catalysed nucleophilic addition of carbonyl and aryl imine compounds *via* hydrazone intermediate using aldehydes as carbanion equivalents.

In 2017, Li and co-workers reported the ruthenium catalysed umpolung strategy for the nucleophilic addition to carbonyl^164^ (Scheme 48a) and aryl imine^165^ (Scheme 48b) compounds through hydrazone intermediates using aldehydes as carbanion equivalents. The unique chemoselectivity exhibited by carbonyl-derived carbanion equivalents is demonstrated by their tolerance to protic reaction media and good functional group compatibility.

**Scheme 49:**
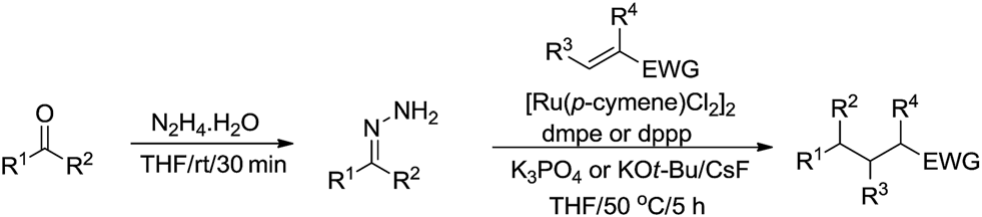
Ruthenium-catalysed conjugate additions *via* hydrazone intermediate.

Subsequently, they described a ruthenium catalysed conjugate additions *via* hydrazone approach, in which water was tolerated in the transformation (Scheme 49).^166^

**Scheme 50:**
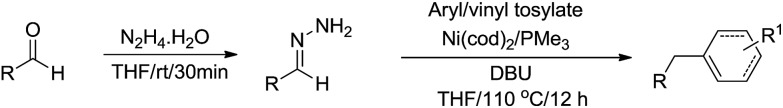
Nickel-catalysed C(sp^2^)–C(sp^3^) cross-coupling reaction *via* hydrazone intermediate.

Recently, Li and coworkers reported a nickel-catalysed C(sp^2^)–C(sp^3^) cross-coupling reaction from two sustainable biomass-based feedstocks: phenol derivatives with umpolung aldehydes through moisture/air-stable hydrazones intermediate generated *in situ* (Scheme 50).^167^ Water tolerance, functional group compatibility and late-stage elaboration of complex biological molecules exemplified its practicability and unique chemoselectivity over stoichiometric organometallic reagents. Further development of such reactions in water is foreseen and being actively pursued in our lab.

## Conclusions

The past few decades have witnessed the rapid development of water-based organic synthesis, in which the organometallic reactions in water has also made considerable progress. This article aims to summarize the key approaches for realizing the various organometallic reactions focusing on nucleophilic additions in aqueous media, which provides the readers with perspectives for further developments in this field. In view of future sustainability, the use of renewable biomass-based feedstocks constitutes an important part for sustainable development of chemical industry. The development of efficient organometallic reactions in water provides important tools for the direct conversion of biomass-derived feedstocks into high-valued chemicals and direct modification of biomolecules under native aqueous environment, and constitutes overall synthetic efficiency.

## Conflicts of interest

The authors declare no competing interests.
